# Semaphorin 4D (CD100) expression as a predictor for the response to induction therapy in pediatric patients with B-acute lymphoblastic leukemia

**DOI:** 10.1007/s00431-026-06915-5

**Published:** 2026-04-17

**Authors:** Rasha A. Elkholy, Doaa El Amrousy, Doaa E. Elgezawy, Reem A. Elkholy, Ola Taha, Abeer A. Eltoukhy, Eman Elaskary

**Affiliations:** 1 Clinical Pathology Department, Faculty of Medicine, Tanta University, Tanta, Egypt; 2 Pediatric Department, Faculty of Medicine, Tanta University, Tanta, Egypt; 3 Pharmacology Department, Faculty of Medicine, Tanta University, Tanta, Egypt; 4 Pharmacology Department, Faculty of Medicine, Badr University in Cairo, Cairo, Egypt

**Keywords:** Semaphorin 4D, Acute lymphoblastic leukemia, Induction therapy, Predictive value

## Abstract

Semaphorin 4D (Sema4D)/(CD100) expression is upregulated in different human cancers, and it is associated with poor prognosis; however, its prognostic value in B-acute lymphoblastic leukemia (B-ALL) remains unclear. We aimed to study the prognostic value of Sema4D expression to predict response to induction therapy in pediatric patients with B-ALL. Sixty newly diagnosed pediatric patients with B-ALL were enrolled, and their peripheral mononuclear cells (PBMCs) were isolated for assessment of Sema4D expression by multiparametric flow cytometry before initiating therapy. Then, we followed up with the patients for their response to induction therapy. Sema4D expression is significantly higher in non-responders to induction therapy than in responders. Using the receiver operating characteristic (ROC) curve, Sema4D expression > 18% significantly identifies patients at risk for induction failure. Logistic regression analyses confirmed that Sema4D expression is significantly associated with poor response to induction therapy.

*Conclusion*: PBMCs’ Sema4D expression can be considered a potential marker for predicting the response to induction therapy in pediatric patients with B-ALL. So, it may serve to optimize treatment decisions for those patients.

**Table:** 

**What is Known:** • *Semaphorin 4D (Sema4D)/(CD100) expression is upregulated in different human cancers, and it is associated with poor prognosis.* • *However, its prognostic value in B-acute lymphoblastic leukemia (B-ALL) in pediatrics remains unclear.*	
**What is New:** • *We aimed to study the prognostic value of Sema4D expression to predict response to induction therapy in pediatric patients with B-ALL.* • *Sema4D expression can be considered a potential marker for predicting the response to induction therapy in pediatric patients with B-ALL. So, it may serve to optimize treatment decisions for those patients.*

**What is Known:**

• *Semaphorin 4D (Sema4D)/(CD100) expression is upregulated in different human cancers, and it is associated with poor prognosis.*

• *However, its prognostic value in B-acute lymphoblastic leukemia (B-ALL) in pediatrics remains unclear.*

**What is New:**

• *We aimed to study the prognostic value of Sema4D expression to predict response to induction therapy in pediatric patients with B-ALL.*

• *Sema4D expression can be considered a potential marker for predicting the response to induction therapy in pediatric patients with B-ALL. So, it may serve to optimize treatment decisions for those patients.*

## Introduction

Acute lymphoblastic leukemia (ALL) is one of the most frequent childhood malignancies, accounting for about one-third of childhood cancers [1]. The cure rates and survival outcomes for pediatric patients with ALL have improved dramatically over the past several decades [2]**.** Improvements are largely due to advances in the understanding of the molecular genetics and pathogenesis of the disease, the incorporation of risk-adapted therapy, the advent of new targeted agents, and the use of allogeneic hematopoietic stem cell transplantation (HSCT) [3]. Despite this improvement, as many as 20% of patients will experience relapse.

On current treatment regimens, the intensity of upfront treatment is stratified based upon prognostic factors to improve cure rates for those at the highest risk of relapse and minimize treatment-related morbidity for lower-risk patients [4]. Therefore, there is a critical need for novel prognostic markers to predict treatment outcomes more effectively and as early as possible.

Semaphorin 4D (Sema4D), also known as CD100, belongs to the family of semaphorins – signaling proteins. It was initially described as an axonal guidance molecule during neuronal development. Sema4D binds to its plexin receptors (PLXN-B1 and -B2), as well as the non-plexin low-affinity receptor CD72 [5, 6]. The effects of Sema4D are mediated by plexins in non-immune tissues (neurons, endothelial cells, tumor cells) and by CD72 in the immune system. In this respect, the plexin-dependent activity of Sema4D is generally associated with the cytoskeleton reorganization and regulation of directed axon/dendrite growth [7, 8], and cell migration [9] while the activity mediated by CD72 is involved in the key events of adaptive immune response, such as antigen activation of B lymphocytes [6], maturation of dendritic cells, and T lymphocyte priming [10].

Transmembrane Sema4D is a 150 kDa glycoprotein expressed in T lymphocytes, B lymphocytes, dendritic cells, macrophages, neutrophils, and eosinophils. It can be converted by proteolytic cleavage into a soluble form (sSema4D) of 120 kDa, that retains the functions of its membrane analogue [11].

Semaphorins’ activities have been extensively studied in many types of solid tumors, such as cutaneous squamous cell carcinoma, head and neck squamous cell carcinoma, lung cancer, breast cancer, pancreatic cancer, soft tissue sarcoma, and others [12]. Tumors overexpressing Sema4D have been indicated to be highly invasive with poor prognosis and therapeutic response [13].

At the level of hematological malignancies, the interaction of Sema4D with its receptor has been revealed to promote survival and inhibit apoptosis in B-chronic lymphocytic leukemia (B-CLL) cells [14]. Also, Sema4D expression was considerably higher in acute myeloblastic leukemia (AML) patients than in healthy controls, and it was linked to risk stratification and poor prognosis [15]. However, previous studies reported higher expression of Sema4D in B-ALL compared to healthy subjects; its impact on patients’ prognosis is still unclear.

Therefore, we aimed to study Sema4D expression and its ability to identify response to induction therapy in pediatric patients with B-ALL.

## Subjects and methods

### Patient recruitment

This prospective cohort study was carried out on 60 children newly diagnosed with B-ALL referred to the Hematology/Oncology Unit, Pediatric Department, Tanta University Hospitals during the period from January 2024 to May 2025.

Inclusion criteria: Pediatric patients newly diagnosed with B-ALL before starting therapy.

Exclusion criteria: age less than 1 year, prior therapy, and relapsed cases.

Cases were diagnosed with B-ALL based upon complete blood count (CBC), bone marrow (BM) examination, as well as immunophenotyping.

All included children underwent thorough history taking and complete physical examination. Routine laboratory investigations such as complete blood count (CBC), C-reactive protein (CRP), and erythrocyte sedimentation rate (ESR).

A cytogenetics study was done on BM samples using conventional karyotyping by staining the cells in the metaphase using standard banding techniques after short-term culture. The description of the karyotypes was done according to the International System for Human Cytogenetic Nomenclature. t (9:22) and t (12:21) were detected by fluorescence in situ hybridization (FISH) technique using commercially available probes; at least 20 metaphases were scanned under a fluorescent microscope.

### Sampling

Sema4D expression was measured in peripheral blood at the time of diagnosis and before starting therapy. Fresh venous blood samples were obtained under complete aseptic precautions via standard venipuncture using Vacuette sodium heparin blood collection tubes (Greiner Bio-One, Austria) for separation of peripheral blood mononuclear cells (PBMCs).

### Separation of mononuclear cells, and staining of Sema4D

**Fig. 1:**
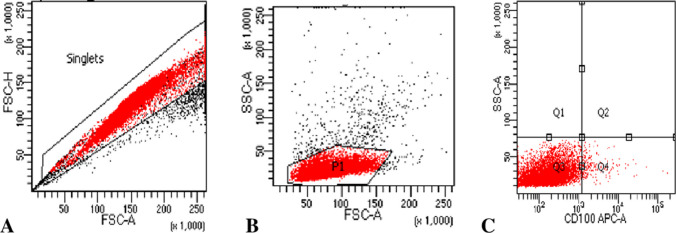
**A** Gating on singlets using FSC-A versus FSC-H to exclude doublets, **B** Gating using FSC-A versus SSC-A on mononuclear cells, **C** negative expression of Sema4D

**Fig. 2:**
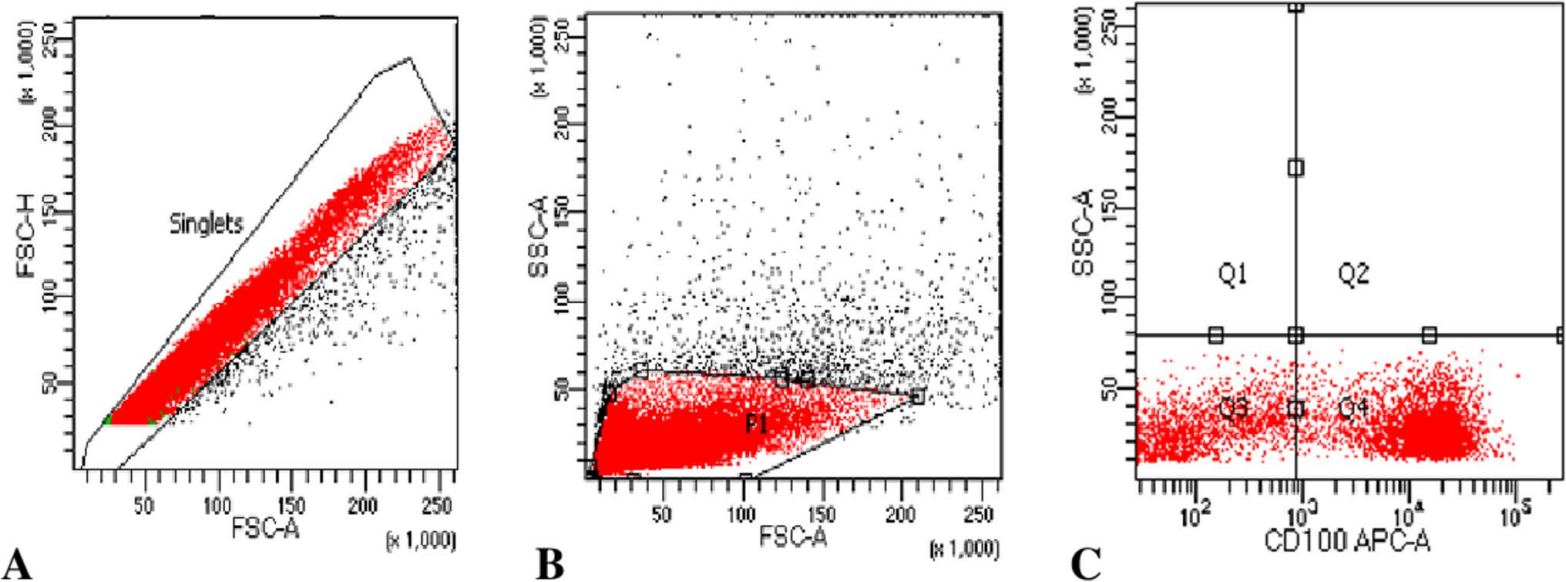
**A** Gating on singlets using FSC-A versus FSC-H to exclude doublets, **B** Gating using FSC-A versus SSC-A on mononuclear cells, **C** Positive expression of Sema4D

Samples were diluted with an equal volume of phosphate buffer saline, then PBMCs were isolated by standard Ficoll-hypaque density gradient centrifugation according to the technique described by Boyum, 1968 [16]. The count was adjusted to a concentration of 1 × 10^6^/ml PBMCs/tube. PBMCs were stained with 5µ of Sema4D using anti-CD100 Alexa Fluor-labeled monoclonal antibody, BD Biosciences, Catalog number 564873, Clone A8, according to the manufacturer’s instructions. The instrument setting was checked by using Cytometer Setup and Tracking (CS&T) beads provided by the manufacturer before sample acquisition. Samples were acquired using a BD FACS Canto™ II eight-color flow cytometer. A total of 50,000 events were collected, and then the data were analyzed using BD FACS Diva software. Gating first to identify singlets and exclude doublets using Forward Scatter Area (FSC-A) versus Forward Scatter Height (FSC-H), then FSC-A versus Side Scatter Area (SSC-A) on PBMCs, the gate includes all PBMCs, leukemic blast cells, as well as non-malignant immune cells, lymphocytes, and monocytes. Positive and negative expression of CD100 are shown in Figs. 1 and 2.

### Follow-up of the patients

After being fully investigated, all the patients received induction chemotherapy according to the Modified St Jude Children’s Research Hospital (SJCRH) Total Therapy XV Protocol [17]. At the end of induction therapy on day 29, all patients were reevaluated by CBC, BM samples, as well as immunophenotyping for assessment of minimal residual disease (MRD). Flowcytometry-based MRD was processed using a single eight colors immunostaining tube as follows: B.M cells count was adjusted to 1 × 10^6^ cells/tube, cells were incubated for 20 min in the dark at room temperature with the appropriate panel of antibodies that include backbone markers CD45, CD19, CD34, CD10, CD20, and CD38, as well as CD58 and CD81. Antibodies were previously titrated to determine the required amounts. After that, lysis was done using BD FACS lysis solution for 15 min, then washed twice using phosphate buffer solution (PBS). Cells were suspended in PBS for acquisition. Acquisition of at least 1 × 10 ^6^ events/tube is required to distinguish leukemic cells from normal B-lineage subpopulations (hematogones) in the B.M during treatment. Sequential gating was done. MRD positivity was defined as the presence of an abnormal blast population comprising at least 0.01% of total cells.

Complete remission was identified by the absence of peripheral blood blasts and BM blast cells less than 5%, with minimal residual disease (MRD) level < 0.01%. Poor response to induction was defined by the reappearance of greater than 5% BM blasts and/or central nervous system (CNS) infiltration by leukemic cells, with MRD level ≥ 0.01%. Those who didn't achieve complete remission received a second induction cycle [17].

## Statistical analysis

Data was analyzed using IBM SPSS software package version 20.0. (Armonk, NY: IBM Corp). Categorical data were represented as numbers and percentages. For continuous data, they were tested for normality by the Kolmogorov–Smirnov test. Quantitative data were expressed as range (minimum and maximum), mean, standard deviation, median, and interquartile range (IQR). The Mann–Whitney test was used to compare two groups with non-normally distributed quantitative variables. The Spearman coefficient test was used to correlate the variables. The receiver operating characteristics (ROC) curve and area under the curve (AUC) were calculated to determine the optimal cutoff values, specificity, sensitivity, and accuracy of CD100 expression. The logistic regression analyses were utilized to identify independent factors influencing response to induction therapy. The level of statistical significance was set at a *p*-value of 0.05.

Effect size for the Mann–Whitney U comparison was estimated using the rank-biserial correlation (r₍rb₎), calculated as r₍rb₎ = 1 − 2U/(n₁n₂). The observed effect size (r₍rb₎ ≈ 0.49) corresponds to a moderate-to-large effect. A post-hoc power assessment was performed using the observed effect size. Given the sample sizes (*n* = 43 responders, *n* = 17 non-responders) and a large observed effect (rank-biserial r ≈ 0.49; corresponding to Cohen’s d ≈ 1.1), the study had adequate power (> 80%) to detect differences of this magnitude.

## Results

### Patients’ characteristics and basic laboratory data

Our prospective study was conducted on 60 children newly diagnosed with B. ALL. There were 37 (61.7%) males and 23 (38.3%) females with a male-to-female ratio of 1.6:1. The age of the patients ranged from 1.5 to 16 years, with a mean of 7.55 ± 3.26.

At diagnosis, the mean and standard deviation (SD) of hemoglobin (Hb) were 7.73 ± 1.84 (gm/dl), blast cells percentage in BM 84.05 ± 12.43 (%), and erythrocyte sedimentation rate (ESR) 80.97 ± 15.60 (mm/h). The median and IQR for the total leucocytic count (TLC) 19.50 (8.0–35.0) × 10^3^/cmm, platelet count 69.50 (37.0–89.0) × 10^3^/cmm, blast cells percentage in P.B 37.50 (20.0–60.0) %, lactate dehydrogenase (LDH) median level was 853.0 (663.0–1238.0) IU/L.

Regarding cytogenetics data, t (9:22) and t (12:21) were positive in 5 (8.3%) and 14(23.3%) patients, respectively. Hyperdiploidy was present in 16 patients (26.7%), while 26 (43.3%) patients exhibited a normal karyotype. Sema4D expression ranged in the studied patients from 3 to 76% with a median (IQR) of 16.50 (9.50–40.0) % (Table 1).

**Table 1:** Basic demographic and laboratory data of the studied patients

Parameters	No. (%)
Sex	
Male	37 (61.7%)
Female	23 (38.3%)
Age	
Min.–Max	1.50–15.0
Mean ± SD	7.55 ± 3.26
Hemoglobin (gm/dl)	
Min.–Max	4.0–12.0
Mean ± SD	7.73 ± 1.84
Total leucocytic count (× 10^3^/cmm)	
Min.–Max	1.0–96.0
Median (IQR)	19.50 (8.0–35.0)
Platelet’s count (× 10^3^/cmm)	
Min.–Max	10.0–185.0
Median (IQR)	69.50 (37.0–89.0)
Blast percentage in peripheral blood	
Min.–Max	0.0–90.0
Median (IQR)	37.50 (20.0–60.0)
Blast percentage in bone marrow	
Min.–Max	50.0–98.0
Mean ± SD	84.05 ± 12.43
Lactate dehydrogenase	
Min.–Max	487.0–2800.0
Median (IQR)	853.0 (663.0–1238.0)
Erythrocyte sedimentation rate	
Min.–Max	45.0–100.0
Mean ± SD	80.97 ± 15.60
t(9:22)	
Negative	55 (91.7%)
Positive	5 (8.3%)
t(12:21)	
Negative	46 (76.7%)
Positive	14 (23.3%)
Hyperdiploidy	
No	44 (73.3%)
Yes	16 (26.7%)
Normal karyotype	
No	34 (56.7%)
Yes	26 (43.3%)
CD100%	
Min.–Max	3.0–76.0
Median (Min.–Max.)	16.50 (9.50–40.0)

*SD* standard deviation, *IQR* inter-quartile range

### Relation between Sema4D expression and age, laboratory data, and response to induction therapy

No significant correlation was observed between Sema4D expression and age and other laboratory data as illustrated in Table 2.

**Table 2:** Correlation between Sema4D expression and different parameters (*n* = 60)

Variables	Sema4D %	
	*r*	*P*
Age (years) Hemoglobin (gm/dl) Total leucocytic count X10^3^ (cmm) Lymphocyte count X10^3^ (cmm)Platelets count X10^3^ (cmm) Blast cells percentage in peripheral blood (%) Blast cells percentage in bone marrow (%) Lactate dehydrogenase (U/L) Erythrocyte sedimentation rate (mm/h)	0.236 − 0.103 0.070 0.121 0.011 − 0.042 0.101 0.169 0.064	0.069 0.433 0.596 0.261 0.935 0.751 0.442 0.195 0.624

*r* Spearman coefficient, *Sema4D* Semaphorin 4D

In addition, there is no significant difference in the expression levels of Sema4D in respect to sex or cytogenetic analysis (t (9;22), t (12:21), normal karyotype, and hyperdiploidy) (*P* = 0.381, 0.294, 0.965, 0.262, and 0.952, respectively) as shown in Table 3.

**Table 3:** Relation between Sema4D expressions and different parameters (*n* = 60)

Parameters	N	Median (Min.–Max.)	*U*	* p*
Sex				
Male Female	**37** **23**	15.0 (3.0–76.0) 18.0 (3.0–70.0)	368.0	0.381
Response to induction				
Poor response Good response	**17** **43**	41.0 (8.0–76.0) 15.0 (3.0–60.0)	188.0^*^	0.004^*^
MRD in BM				
Positive ≥ 0.01% Negative < 0.01%	**17** **43**	41.0 (8.0–76.0) 15.0 (3.0–60.0)	188.0^*^	0.004^*^
t (9:22)				
Negative Positive	**55** **5**	15.0 (3.0 – 76.0) 30.0 (15.0 – 50.0)	97.50	0.294
Hyperdiploidy No Yes	**44** **16**	18.0 (4.0–70.0) 11.0 (3.0–76.0)	285.0	0.262
t (12:21)				
Negative Positive	**46** **14**	16.50 (3.0 – 76.0) 26.50 (4.0 – 50.0)	319.50	0.965
Hyperdiploidy				
No Yes	**44** **16**	18.0 (4.0 – 70.0) 11.0 (3.0 – 76.0)	285.0	0.262
Normal Karyotype				
No Yes	34 26	16.50 (3.0 – 76.0) 16.50 (5.0 – 70.0)	438.0	0.952

*U* Mann–Whitney, *MRD* minimal residual disease, *BM* bone marrow

^*^Statistically significant at *p* ≤ 0.05

After the end of induction therapy, complete remission was achieved by 43/60 (71.6%) cases, with MRD negativity in B.M (< 0.01%), where the remaining 17 cases (28.3%) were refractory to the first induction cycle with MRD in B.M ≥ 0.01%.

**Fig. 3:**
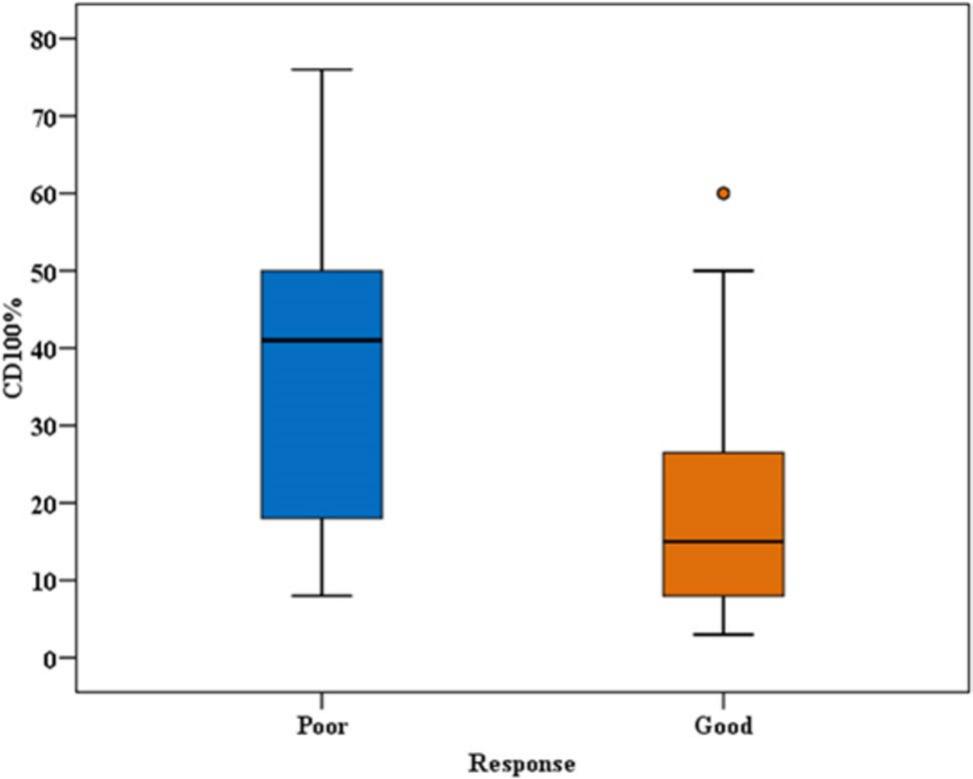
Expression of Sema4D (CD100) % in good and poor responders to induction therapy

Sema4D expression ranged from 3.0 to 60.0% with a median of 15% in responders and ranged from 8.0 to 76% with a median of 41% in the non-responders’ group. The difference between Sema4D expressions regarding the response to therapy was highly significant (*p*-value < 0.004) (Fig. 3).

### Diagnostic performance of Sema4D expression to differentiate good responders to induction therapy from poor responders

**Fig. 4:**
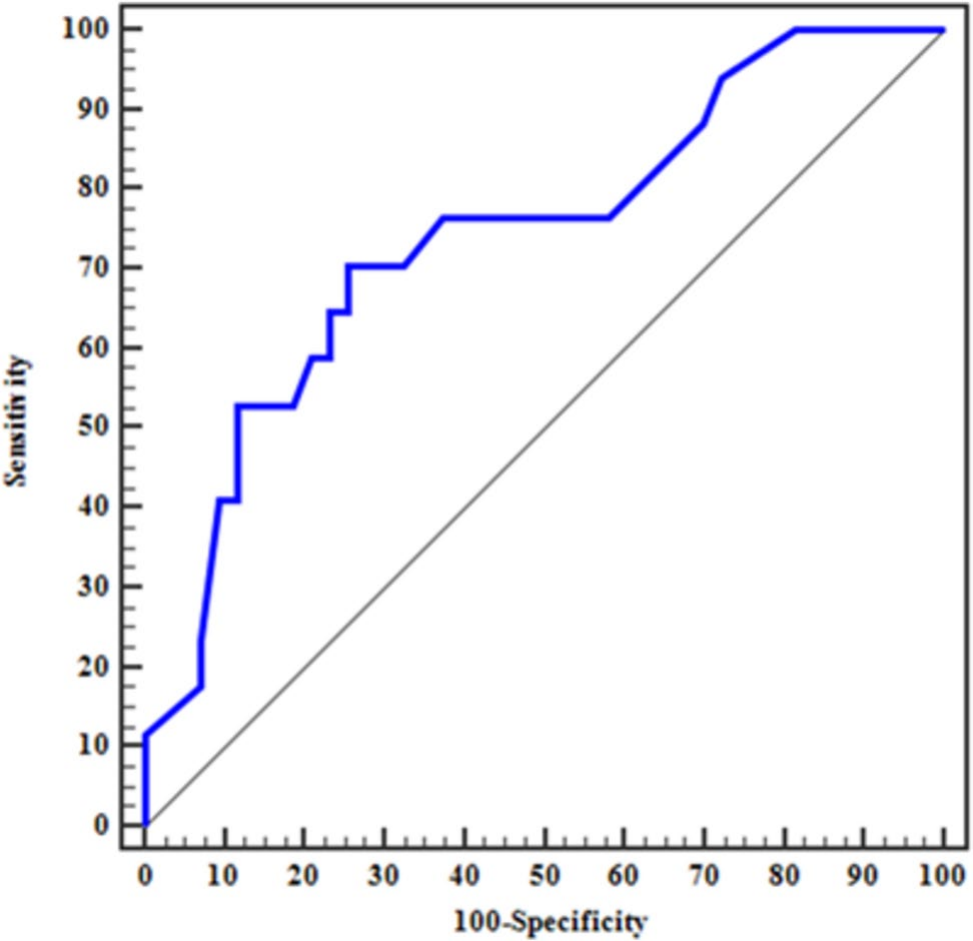
ROC curve for Sema4D (CD100) % to predict poor response (*n* = 17) from good response (*n* = 43), AUC 95% CI (0.601–0.885)

ROC curve analysis was performed to determine the best cutoff of Sema4D expression that predicts poor response to induction therapy and showed that the best cutoff value was > 18% yielding a sensitivity of 70.59% with an AUC of 0.743, 95% confidence interval (C.I) 0.601–0.885, *p*-value 0.004 (Fig. 4).

To determine the related covariables that may be associated with poor response to induction therapy in B.ALL, logistic regression analysis was performed and revealed that Sema4D expression is significantly associated with poor response to induction therapy in both univariate and multivariate models, with a correlation matrix < 0.007 (Table 4).

**Table 4:** Univariate and multivariate logistic regression analysis for the parameters affecting poor response

Parameters	Univariate analysis		Multivariate analysis	
	P	OR (LL–UL 95% CI)	p	OR (LL–UL 95% CI)
Sex (female versus male)	**0.776**	1.181 (0.375–3.719)		
Age (> 8.0 versus ≤ 8 years)	**0.269**	1.898 (0.610–5.909)		
Hemoglobin (> 7.65 versus ≤ 7.65 gm/dl)	**0.392**	0.609 (0.195–1.897)		
Total leucocytic count (> 19.5 versus ≤ 19.5 × 10^3^/cmm)	**0.051**	3.333 (0.998–11.139)	**0.800**	1.226 (0.253–5.933)
Platelets count (> 69.50 versus ≤ 69.50 × 10^3^/cmm)	**0.392**	1.643 (0.527–5.120)		
Peripheral blood blast cells (37.50 versus ≤ 37.50%)	**0.157**	2.316 (0.724–7.407)	**0.119**	3.743 (0.713–19.643)
Bone marrow blast cells (> 90 versus ≤ 90%)	**0.239**	0.504 (0.161–1.576)		
Lactate dehydrogenase (> 853.0 versus ≤ 853.0 IU/L)	**0.392**	1.643 (0.527–5.120)		
Erythrocyte sedimentation rate (> 80.0 versus ≤ 80.0 mm/h)	**0.309**	1.805 (0.578–5.631)		
t(9:22) (positive versus negative)	**0.125**	4.393 (0.664–29.056)	**0.115**	6.138 (0.643–58.591)
t(12:21) (positive versus negative)	**0.982**	1.015 (0.270–3.821)		
Hyperdiploidy (present versus absent)	**0.326**	0.495 (0.121–2.019)		
Normal karyotype (yes versus no)	**0.832**	0.884 (0.283–2.759)		
Sema4D (CD100%) (> 18% versus ≤ 18%)	0.010^*^	4.971 (1.464–16.887)	0.015^*^	5.664 (1.392–23.058)

*OR* odds ratio, *CI* confidence interval, *LL* lower limit, *UL* upper Limit

^*^Statistically significant at *p* ≤ 0.05

## Discussion

Despite the favorable survival rates in the B-ALL pediatric population, a significant number of patients develop resistance to therapy, resulting in poor prognosis [18]. Thus, identification of new biomarkers predicting response to treatment will lead to better treatment protocol decision-making and monitoring of the course of the disease.

Multiparametric flow cytometry immunophenotyping is considered a potent technology used to identify lineage assignment, phenotypic aberrations, and expression of therapeutic targets by the malignant leukemic cells [19]. The identification of surface antigens is essential for the assignment of the proper treatment plan and is also valuable for assessing prognosis and searching for applicable markers to detect minimal residual disease [20].

Although Sema4D expression has been previously studied in various human cancers, including acute leukemia, data on its prognostic relevance in acute lymphoblastic leukemia are still inadequate.

This study’s principal aim was to evaluate the expression of Sema4D in pediatric patients with B-ALL and its influence on the response to induction therapy for the first time. For this reason, sixty pediatric patients newly diagnosed with B-ALL were enrolled in this study, and their Sema4D expression was detected by flow cytometry on their PBMCs after separation. Sema4D expression ranged in the studied patients from 3 to 76% with a median (IQR) of 16.50 (9.50–40.0) %. This was in agreement with Xue et al. [21], and Jiang et al. [22], who reported that Sema4D was highly expressed in PBMCs of acute leukemia patients.

Regarding the correlation between Sema4D expressions and different clinical and laboratory parameters, our results revealed that there is no statistically significant correlation observed between Sema4D expression with age and laboratory data. Also, no statistically significant difference was reported regarding Sema4D expression regarding gender or cytogenetic data. These results were kept on track with Xue et al. [21] who reported that Sema4D expression in PBMCs of ALL and AML patients and bone marrow mononuclear cells (BMMCs) of ALL patients was not correlated with age, risk classification, extramedullary infiltration, chromosome translocation, and fusion genes**,** while soluble Sema4D levels were significantly correlated with bone marrow leucocyte count and blast count, but not with peripheral blood blast count. To our knowledge, this is the first study that report the impact of Sema4D flow-cytometric expression on the response to induction therapy in B-ALL. Therefore, we established a cutoff value of > 18% as calculated by the ROC curve to identify patients who are at risk for induction failure that may require intensive therapy from the start to improve outcome. After that, logistic regression analysis was performed to determine the related covariables that may associate with poor response to induction therapy in pediatric B-ALL patients including Sema4D PBMCs expression, and we reported that Sema4D expression was statistically significantly affecting the response to induction therapy with odds ratio 1.049 (1.016- 1.082) and 5.664 (1.392–23.058) and *p*-value 0.003 and 0.015 in both univariate and multivariate regression analysis respectively.

Previous results by Jiang et al. [22] demonstrated that Sema4D expression may be implicated in B-ALL pathogenesis as Sema4D knockdown induced cell cycle arrest in the G0/G1 phase, increased apoptosis, and inhibited proliferation; on the contrary, overexpression of Sema4D promoted cell division and proliferation and inhibited apoptosis. In Jurkat cells, Sema4D knockdown inhibited proliferation and promoted apoptosis, while Sema4D overexpression resulted in a decreased abundance of cells in the G0/G1 phase and promoted proliferation and promoted the migratory ability of Jurkat cells and the invasive ability of B-ALL-1 cells. These results on the effect of Sema4D on invasion and migration are consistent with a previous study in breast cancer [23].

Moreover, Sema4D has been indicated to phosphorylate tyrosine kinase receptors [protein-tyrosine kinase 2-beta (Pyk2) or Src] and ERK1/2, and phosphorylated Pyk2 and Src further activate the PI3K/AKT signaling pathway and mediate cell invasion and migration. As the activation of PI3K, AKT, and ERK is important in regulating cell proliferation, invasion, migration and apoptosis [24–26], it is speculated that Sema4D promoted leukemia development via activating the PI3K/AKT and ERK pathways.

Our in vivo findings that elevated Sema4D expression predicts poor response to induction therapy are biologically plausible in light of previously reported in vitro data. Sema4D has been shown to activate the PI3K/AKT and ERK signaling pathways, which are central mediators of leukemic cell survival and resistance to apoptosis. Since induction chemotherapy in pediatric B-ALL primarily exerts its therapeutic effect through apoptosis induction, activation of these survival pathways may attenuate cytotoxic efficacy. Experimental studies have demonstrated that Sema4D overexpression promotes cell-cycle progression and inhibits apoptosis, whereas Sema4D knockdown induces G0/G1 arrest and enhances apoptotic signaling [24–26]. Therefore, high Sema4D expression may contribute mechanistically to chemoresistance, explaining the poorer induction response observed in our cohort.

These results showed the potential ability of Sema4D expression to promote resistance to chemotherapy by resisting apoptosis, and predict response to induction therapy early, and determine those who may need a more aggressive therapy protocol from the start. From a clinical perspective, Sema4D expression assessed at diagnosis may provide additional value for risk stratification. While MRD remains the strongest prognostic marker in pediatric B-ALL, it reflects early treatment response rather than baseline disease biology. In contrast, Sema4D expression can be measured before therapy initiation and may identify patients with intrinsic resistance potential. A cutoff value of > 18% could therefore serve as an early warning marker to prompt closer MRD monitoring, consideration of intensified induction strategies, or future incorporation into composite risk models alongside cytogenetics and molecular markers. However, prospective multicenter validation is necessary before routine clinical implementation.

In addition to its role as a prognostic biomarker, Sema4D may represent a potential therapeutic target in pediatric B-ALL. Given its involvement in the activation of PI3K/AKT and ERK signaling pathways, pharmacologic inhibition of Sema4D or its downstream effectors could enhance chemosensitivity and improve treatment outcomes. Targeting molecules that contribute to leukemic cell survival and resistance mechanisms has become an important strategy in modern hematologic oncology. If future studies confirm the pathogenic role of Sema4D in mediating apoptosis resistance and disease persistence, therapeutic modulation of this pathway, either directly through monoclonal antibodies or indirectly via pathway inhibitors, may provide a novel adjunct to conventional chemotherapy protocols.

Limitations of the study: Despite the promising findings, our study has certain limitations**.** The relatively small sample size, short follow-up, and a lack of an external validation cohort reflect the single-center nature of the study, which may limit the generalizability to broader pediatric B-ALL populations. However, the observed effect size (r₍rb₎ ≈ 0.49) corresponds to a moderate-to-large effect, indicating that the study was sufficiently powered to detect biologically meaningful differences despite the group imbalance.

Measurement of Sema4D expression on PBMCS, that include non-malignant immune cells, is another limitation. Moreover, the association between Sema4D surface expression and its soluble level was not investigated. Therefore, larger multicenter studies with extended follow-up to determine long-term outcomes, measurement of sSema4D to identify if shedding dynamics and receptor-mediated feedback can indirectly influence how much membrane Sema4D is present, as well as mechanistic investigations, are needed to validate our findings and to determine whether incorporating Sema4D expression into current risk stratification systems can improve clinical decision-making and patient outcomes.

## Conclusion

PBMCs' Sema4D expression can be considered a potential biomarker for predicting the response to induction therapy in pediatric patients with B-ALL. So, it may serve to optimize treatment decisions for those patients.

## Data Availability

All the data of the study are available from the corresponding author on reasonable request.

## Abbreviations

ALL: Acute lymphoblastic leukemia
AML: Acute myeloblastic leukemia
AUC: Area under the curve
B-CLL: B-chronic lymphocytic leukemia
BM: Bone marrow
CBC: Complete blood count
CNS: Central nervous system
CRP: C-reactive protein
ESR: Erythrocyte sedimentation rate
Hb: Hemoglobin
HSCT: Hematopoietic stem cell transplantation
IQR: Inter-quartile range
MRD: Minimal residual disease
PBMCs: Peripheral blood mononuclear cells
ROC: Receiver operating characteristics
SD: Standard deviation
TLC: Total leucocyte count
